# Isolation of *Candida auris* from invasive and non-invasive samples of a patient suffering from vascular disease, Italy, July 2019

**DOI:** 10.2807/1560-7917.ES.2019.24.37.1900549

**Published:** 2019-09-12

**Authors:** Francesca Crea, Giulia Codda, Andrea Orsi, Alberto Battaglini, Daniele Roberto Giacobbe, Emanuele Delfino, Riccardo Ungaro, Anna Marchese

**Affiliations:** 1 Unità Operativa di Microbiologia, Ospedale Policlinico San Martino-IRCCS, Genoa, Italy; 2 FC and GC contributed equally to this article; 3 Microbiology Unit, DISC University of Genoa, Genoa, Italy; 4 Unità Operativa di Igiene, University of Genoa (DISSAL) and Ospedale Policlinico San Martino-IRCCS, Genoa, Italy; 5 Unità Operativa Clinica Malattie Infettive, University of Genoa (DISSAL) and Ospedale Policlinico San Martino-IRCCS, Genoa, Italy; 6 Unità Operativa di Microbiologia, University of Genoa (DISC) and Ospedale Policlinico San Martino-IRCCS, Genoa, Italy

**Keywords:** Candida, antifungal resistance, fungal infection, epidemic clone, MDR, epidemiology

## Abstract

We recently isolated *Candida auris* from a blood culture and cutaneous swabs of a patient in her mid-70s. Our routine phenotypic methods failed to identify the microorganism, but it was identified by molecular tests and MALDI-TOF MS analysis. Our report, the first from Italy, further underlines the geographically wide distribution of *C. auris* and the need to confirm species identification of any suspicious colony as soon as possible to stop its spread.

*Candida auris* is a rapidly spreading, multidrug-resistant, healthcare-associated pathogen [1].

Since the first description of this novel *Candida* species, isolated from a patient in a Japanese hospital in 2009, the emergence of *C. auris* has been documented on all continents [2]. Invasive infections have been associated with high rates of treatment failure and mortality ranging from 30% to 72% [3]. *C. auris* is thus considered to pose a serious global public health threat.

*C. auris* can colonise patients for a long time, as well as persist on surfaces in healthcare environments [4]. Furthermore, *C. auris* can be misidentified in the diagnostic laboratory when traditional phenotypic methods are used. These features of *C. auris* contribute to its spread in healthcare facilities.

Here, we report a case of *C. auris* being isolated from a female in her mid-70s suffering from vascular disease.

## Case report

On 16 June 2019 (day 1), the patient who had a history of hypertension and dyslipidaemia, was admitted to hospital for endovascular repair of an abdominal aortic aneurysm and left renal artery stenting. Because of surgical complications she was transferred to the intensive care unit (ICU). The postoperative course was further complicated and required left subclavian artery stenting on day 14. The patient first developed a fever on day 17, the same day the patient was transferred to vascular surgery department from the ICU. A computed tomography performed on day 26 for worsening condition and respiratory symptoms showed bilateral ground glass opacities. The patient was then retransferred to the ICU on day 26. Blood cultures were repeatedly collected during intermittent fever episodes that were unresponsive to antimicrobial treatment with meropenem and linezolid. Serum (1,3)-beta-D-glucan tests were performed repeatedly during fever episodes and were constantly negative. Ultimately, *C. auris* grew from blood cultures collected on day 31. The patient was immediately transferred to a single room in the infectious disease unit. *C. auris* also grew from axillary and ear swabs collected on day 41 and day 47 respectively. Treatment with caspofungin was started and all subsequent blood cultures and swabs were negative. On day 39, the patient’s improved clinical condition led to being discharged from the ICU and being admitted to the infectious diseases ward for completion of the antifungal treatment, i.e. until 14 days after the first negative blood culture. At the time of this communication, the patient is still hospitalised in the infectious diseases ward and in stable condition.

## Identification of *Candida auris* and phylogenetic analysis

The pathogen was not identified by VITEK2 Advanced Expert System software version 8.01 (bioMérieux, Macy-l'Étoile, France), reporting a low discrimination between *C. guillermondi* (50%) and *Cryptococcus laurentii* (50%). It was also not identified by MALDI-TOF mass spectrometry (MALDI-TOF MS) analysis using VITEK MS software version 3 (bioMérieux). The microorganism was identified as *C. auris* by a species-specific PCR for GPI protein-encoding genes [5]. MALDI-TOF MS analysis using BD MALDI Biotyper System (Bruker Daltonics, Bremen, Germany) identified the yeast as *C. auris* (score 2.7). This result was further confirmed by D1/D2 region and internal transcribed spacer (ITS) sequencing [6,7]. The BLAST tool at the National Center for Biotechnology Information (NCBI) database was used to perform sequences similarity searches. Our sequences (GenBank accession numbers MN275234 and MN294701) showed > 99% homology with *C. auris*.

The D1/D2 sequences were aligned with the ClustalW programme [8].

A neighbour-joining tree based on 26S rRNA gene D1/D2 domains sequences was generated using MEGA software version X [9].

Phylogenetic analyses showed that our isolate, *C. auris* FG_GE01, clustered with the southern Asian strains (Figure).

**Figure:**
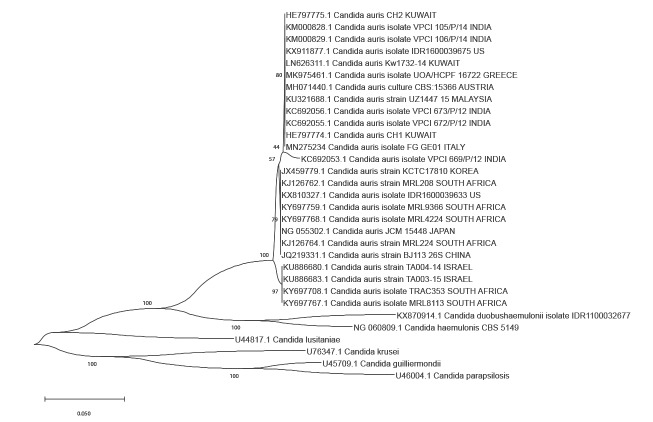
Dendrogram of *Candida auris* with other related *Candida* species, July 2019

MEGAX software was used to align 26S rRNA gene D1/D2 domains sequences and to draw the bootstrap (550 replicates) unweighted pair group method. The tree is drawn to scale with branch lengths in the same units as those of the evolutionary distances used to infer the phylogenetic tree. The Kimura 2-parameter method was used to compute the evolutionary distances.

## Antifungal susceptibility

Antifungal susceptibility was determined using the Clinical and Laboratory Standards Institute (CLSI) microdilution method [10] and Sensititre YeastOne (Thermo Scientific, Waltham, Massachusetts, United States (US)) The following minimum inhibitory concentration (MIC) values were observed: > 256 mg/L (fluconazole), 2mg/L (amphotericin B), 0.5 mg/L (flucytosine), 4 mg/L (voriconazole), 0.25mg/L (posaconazole), 0.5 mg/L (itraconazole), 0.12 mg/L (micafungin), 0.25 mg/L (anidulafungin) and 0.12 mg/L (caspofungin).

## Epidemiological and environmental investigations

Preliminary epidemiological and environmental investigations have not yet revealed the source of the infection. The patient had no history of recent travel abroad or hospital admission and to date, *C. auris* has not been isolated from any of the case’s close contacts. A close contact was defined as a patient who was hospitalised in the same room during the same period (at least 12 hours) of the case or a patient who occupied the same bed as the case immediately after. No contacts outside the hospital, e.g. family members, were swabbed.

In August 2019, screening was performed of the environment and medical devices of the vascular ward, ICU and operating theatre where the patient stayed during the course of hospitalisation. The 36 environmental samples tested (dynamic mattresses, n = 5; bedrails, n = 10; trolley, n = 5; ventilators, n = 5; suction apparatus, n = 5; floor, n = 2; washbasin, n = 2; bed bell, n = 2) were negative. Additional cleaning using hydrogen peroxide and hypochlorite was implemented across the hospital.

## Discussion

To our knowledge, this is the first isolation of *C. auris* in Italy. However, this is not particularly surprising given the widespread distribution of this pathogen and its previous detection in several nearby European countries, including France, Spain and Greece [11].

We were able to correctly identify the microorganism by using MALDI-TOF as well as molecular techniques, approaches that are still not available in many diagnostic laboratories. The first finding of *C. auris* in Italy should encourage a careful approach to yeast identification when non-*albicans Candida* strains are detected. Given the present scenario, confirmation of species identification of any suspicious colony is clearly essential.

Four major phylogenetically distinct clades of *C. auris* have been described: clade I (South Asian), clade II (East Asian), clade III (African) and clade IV (South American) [12]. A potential fifth clade has recently been reported in Iran [13].

The isolate in this study clusters with the southern Asian strains. Clade I has been already described in European hospitals and in hospitals in the US, and linked to outbreaks with invasive infections [14].

Since the potential source of the infection has not been identified, we currently have no basis for any speculation about the origin of *C. auris* in the hospital. The prompt isolation of the patient seems to have stopped the spread. Although specific breakpoints for *C. auris* have not been defined by international committees, susceptibility data published to date suggests that this pathogen exhibits resistance to fluconazole (MICs > 32 mg/L) and different level of susceptibility to the other azoles, echinocandins and amphotericin B. A considerable percentage of *C. auris* strains investigated had high MICs for voriconazole and amphotericin B (MICs > 1). In general, our susceptibility results are in agreement with previous observations [15]. Adopting CLSI breakpoints for closely related species (*C.guillermondi* and *C. parapsilosis*), *C. auris* FG_GE01 was categorised as susceptible to echinocandins. This finding is supported by clinical data: caspofungin treatment was effective and blood samples collected 7 days after starting the treatment were negative. However, development of resistance to echinocandins has also been described in *C. auri*s and other *Candida* species [16,17].

This isolation of *C. auris* is further confirmation its intercontinental distribution, and that judicious use of antifungals coupled with strengthened infection control measures are needed to prevent and control the spread of *C. auris*.
